# Bronchial Wall T2-Weighted MRI Signal: An Emerging Investigational Non-Ionizing Imaging Biomarker in Severe Asthma

**DOI:** 10.3390/biomedicines14091920

**Published:** 2026-08-27

**Authors:** Guido Marchi, Santi Nolasco, Corrado Pelaia, Salvatore Claudio Fanni, Fabrizio Luppi, Michele Mondoni

**Affiliations:** 1 Pulmonology Unit, Cardiothoracic and Vascular Department, University Hospital of Pisa, 56124 Pisa, Italy; 2 Respiratory Medicine Unit, Policlinico “G. Rodolico-San Marco” University Hospital, 95123 Catania, Italy; 3 Department of Health Sciences, University “Magna Græcia” of Catanzaro, 88100 Catanzaro, Italy; 4 Academic Radiology, Department of Translational Research, University of Pisa, 56126 Pisa, Italy; 5 Division of Respiratory Disease, Fondazione IRCCS San Gerardo dei Tintori, 20900 Monza, Italy; 6 Respiratory Diseases Unit, Department of Medicine and Surgery, School of Medicine and Surgery, University of Milano-Bicocca, 20900 Monza, Italy; 7 Respiratory Unit, ASST Santi Paolo e Carlo, Department of Health Sciences, Università degli Studi di Milano, 20146 Milan, Italy

**Keywords:** severe asthma, magnetic resonance imaging, bronchial wall imaging, T2-weighted MRI, ultrashort echo time MRI, airway inflammation, airway remodelling, imaging biomarkers, type 2 inflammation, precision medicine

## Abstract

Severe asthma represents a major clinical challenge, accounting for disproportionate healthcare costs and morbidity despite affecting only 3–5% of patients with asthma. Traditional computed tomography (CT) can quantify bronchial wall thickening and mucus plugging, but concerns about cumulative radiation exposure limit its use for longitudinal monitoring, particularly in patients requiring frequent assessment. Recent advances in magnetic resonance imaging (MRI), specifically T2-weighted sequences combined with ultrashort echo time (UTE) imaging, have enabled quantitative assessment of bronchial wall signal intensity without ionizing radiation. Pilot studies demonstrate that bronchial wall T2-weighted MRI signal intensity (BrWall_T2-MIS) is significantly elevated in severe versus non-severe asthma, correlates with airflow obstruction and type-2 inflammatory markers, and has shown preliminary ability to predict exacerbation risk. Importantly, this biomarker may capture different pathophysiological information than CT-derived wall thickness measurements, potentially reflecting active inflammation rather than fixed remodelling. Although evidence remains preliminary and limited to single-centre studies with small sample sizes, BrWall_T2-MIS represents a promising investigational tool that could enhance phenotyping and therapeutic monitoring in the era of targeted biological therapies for severe asthma.

## 1. Introduction

Asthma affects an estimated 300 million people worldwide [1]. Within this population, severe asthma represents a small but disproportionately burdensome subset, most recently estimated at around 3.7% of patients [1], with per-patient healthcare costs reported to exceed those of type 2 diabetes, stroke, or Chronic obstructive pulmonary disease (COPD), and severe uncontrolled disease alone accounting for over 60% of total asthma-related costs [1,2]—driven substantially by exacerbations, hospitalisations, and cumulative oral corticosteroid toxicity. Despite guideline-driven advances in phenotyping and biological therapies, objective, repeatable tools to characterize airway pathology and monitor treatment response remain limited in routine practice [3].

Severe asthma pathophysiology involves airway inflammation and structural remodelling, both contributing to bronchial wall thickening on imaging [4]. Computed tomography (CT) has been validated for quantifying bronchial dimensions, with measurements correlating with disease severity, lung function, and tissue markers of remodelling [5,6,7]. In addition, chest CT is used to assess airway mucus plugging presence. Mucus plugging has been associated with airflow obstruction [8], ventilation defects assessed with Hyperpolarized xenon-129 magnetic resonance imaging (^129^Xe MRI) [9] and airway remodelling [10]. However, radiation exposure restricts repeated monitoring, crucial especially to assess mucus burden before and after intervention. This is an important constraint in severe asthma where lifelong surveillance and ongoing therapeutic adjustment are often required [11].

Magnetic resonance imaging (MRI) offers high soft tissue contrast without radiation. Recent innovations, including ultrashort echo time (UTE) sequences achieving submillimeter resolution, have mitigated key limitations of lung MRI—particularly rapid T2 signal decay and susceptibility-related signal loss at air–tissue interfaces [12]. T2-weighted imaging, sensitive to tissue water content and inflammation, has proven useful in other respiratory conditions like cystic fibrosis [13,14].

**Figure 1:**
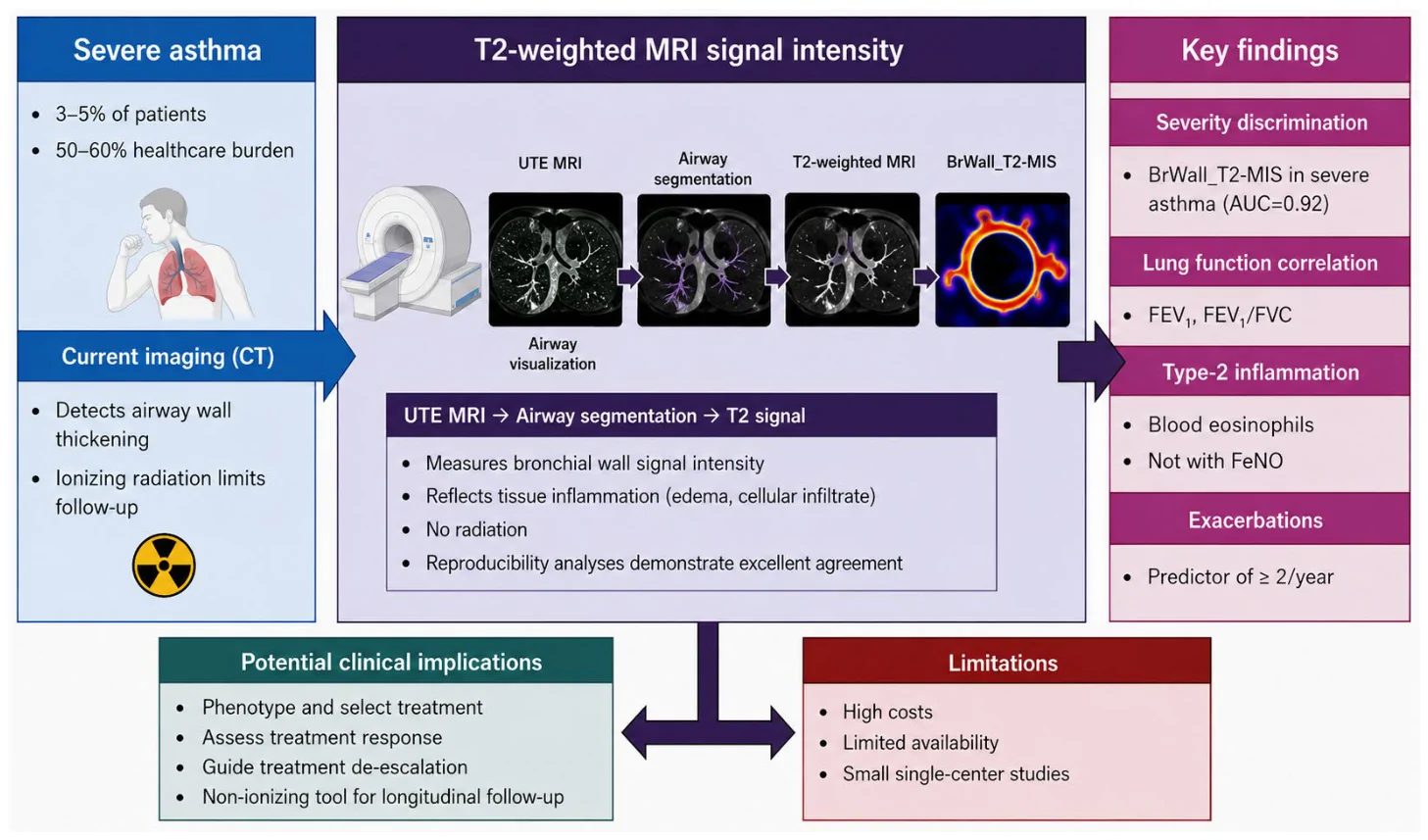
Overview of bronchial wall T2-weighted MRI signal intensity (BrWall_T2-MIS) as an emerging imaging biomarker in severe asthma. The figure illustrates the clinical rationale, technical workflow (UTE MRI → airway segmentation → T2 signal quantification), key findings across four domains (severity discrimination, lung function correlation, type-2 inflammation, and exacerbation prediction), potential clinical implications, and current limitations. Illustrative imaging panels are intended for conceptual and educational purposes to facilitate understanding of the imaging workflow and representative radiological features, and should not be interpreted as diagnostic or quantitative imaging examples. AUC, area under the curve; CT, computed tomography; FeNO, fractional exhaled nitric oxide; FEV_1_, forced expiratory volume in 1 s; FVC, forced vital capacity; MRI, magnetic resonance imaging; UTE, ultrashort echo time.

This viewpoint examines emerging evidence for bronchial wall T2-weighted MRI signal intensity as a potential biomarker in severe asthma, its relationship to established parameters, current limitations, and future directions (Figure 1).

## 2. Technical Foundations

Quantification requires integrating two MRI sequences: three-dimensional UTE imaging for structural airway visualization, and T2-weighted imaging (using MRI sequences designed to be robust to respiratory motion, improving image quality in non-breath-hold conditions) for signal assessment [15,16]. Both are acquired at end-expiration with respiratory triggering.

The workflow involves: bronchial wall segmentation on CT or UTE-MRI; elastic registration aligning T2-weighted with structural images; transferring bronchial masks to registered T2-weighted images; and calculating mean signal intensity within wall regions [15,16]. Signal normalization is essential, typically by centering values relative to whole-image statistics.

This semi-automated approach combines superior spatial resolution of CT/UTE-MRI for airway delineation with T2-weighted imaging’s sensitivity to inflammatory changes. Reported acquisition parameters illustrate this resolution gradient: an isotropic voxel size of 0.625 mm^3^ for CT, 1 mm^3^ for UTE-MRI, and a coarser 1.4 × 1.4 × 3 mm^3^ for the T2-weighted BLADE (PROPELLER-equivalent) sequence used for signal quantification. Reproducibility analyses demonstrate excellent agreement (intraclass correlation coefficient > 0.95) [15].

Several practical aspects remain uncharacterized, including computational time and near-real-time feasibility of the semi-automated pipeline, cross-scanner/vendor reproducibility of signal normalization, and acquisition feasibility during severe airflow limitation or acute exacerbation.

## 3. Practical Aspects of UTE-MRI Versus CT Acquisition

Beyond spatial resolution, the two modalities differ substantially in their practical implementation. CT acquisition is completed within a single short breath-hold at end-normal expiration, typically lasting only a few seconds, and requires no contrast administration. The MRI protocol described [15,16], by contrast, combines two sequential acquisitions—3D UTE imaging (approximately 6–7 min) and T2-weighted BLADE imaging (approximately 4–8 min)—both performed in free-breathing with respiratory-navigator triggering at end-normal expiration, avoiding the need for breath-holding but extending total scan-room time to roughly 10–15 min of acquisition alone, before accounting for patient positioning and coil setup. Neither modality requires contrast administration in the protocols reported to date.

This longer, free-breathing MRI protocol may be better tolerated by patients unable to sustain a CT breath-hold, but it also requires the patient to remain still within the scanner for a more extended period, and is subject to the standard practical constraints of MRI more broadly (safety screening for ferromagnetic implants, and tolerability of the supine, enclosed scanner environment, which may be relevant in patients with severe dyspnoea or claustrophobia). Finally, availability differs markedly between the two modalities: CT scanners are near-universally available, whereas the UTE and T2w-BLADE/PROPELLER sequences used in the studies reviewed here are vendor-specific prototype sequences rather than standard clinical packages, restricting their use to centres with the relevant hardware, software, and technical expertise.

A comparison of the key technical, practical, and clinical characteristics of CT and UTE-MRI is provided in Table 1.

## 4. Current Evidence: What Do We Know?

### 4.1. Differentiation of Severe from Non-Severe Asthma

The pivotal 2025 study by Benlala et al. represents the first systematic evaluation of quantitative bronchial wall T2-weighted signal in asthma phenotyping [15]. In a carefully matched cohort of 15 severe and 15 non-severe asthmatic patients, BrWall_T2-MIS was significantly elevated in the severe asthma group (mean 74 ± 12 versus 49 ± 14 arbitrary units; *p* < 0.001). Receiver operating characteristic (ROC) analysis, using the ATS/ERS Task Force clinical classification of asthma severity as the reference standard, revealed excellent discriminatory ability, with an area under the curve of 0.92 (95% CI: 0.76–0.99) at an optimal BrWall_T2-MIS cutoff of 57 arbitrary units, achieving 100% sensitivity and 80% specificity at that threshold.

Airway structural measurements are increasingly necessary in severe asthma. The biomarkers most commonly propagated in routine care—blood eosinophils and fractional exhaled nitric oxide (FeNO)—have important limitations, both for accurately capturing disease severity and for predicting treatment response [17]. For these reasons, the findings are particularly noteworthy: in this cohort, conventional biomarkers—FeNO and blood eosinophil counts—did not differ significantly between groups, implying that BrWall_T2-MIS captures clinically relevant pathophysiology that is not reflected by standard type 2 inflammatory markers alone [15].

### 4.2. Correlation with Clinical Outcomes

BrWall_T2-MIS demonstrates moderate inverse correlations with pulmonary function parameters across the entire asthma spectrum. Specifically, signal intensity correlates negatively with forced expiratory volume in 1 s (FEV_1_) as percentage predicted (r = −0.54, *p* < 0.001) and FEV_1_/forced vital capacity ratio (r = −0.44, *p* = 0.01) [15]. These associations persist within the severe asthma subgroup, whereas correlations in non-severe asthma are non-significant, suggesting that elevated T2 signal may be particularly relevant in advanced disease characterized by more persistent airflow limitation.

Importantly, patients with chronic airflow obstruction (FEV_1_ < 80% predicted) exhibited significantly higher BrWall_T2-MIS compared to those with intermittent obstruction (77 ± 16 versus 54 ± 15; *p* < 0.001), supporting the hypothesis that bronchial T2 signal reflects disease burden associated with irreversible or poorly reversible airway changes or chronic inflammation [15].

### 4.3. Association with Type-2 Inflammation

Within the severe asthma cohort, BrWall_T2-MIS showed a moderate positive correlation with blood eosinophil counts (r = 0.52, *p* = 0.047), a well-established biomarker of type-2 airway inflammation [15].

Interestingly, no significant correlation was observed with FeNO, likely reflecting the context-dependent relationship between FeNO and eosinophilic airway inflammation, as well as FeNO’s high intra-individual variability [18]. These findings suggest that bronchial wall T2 signal may serve as a potential tissue-level correlate of eosinophilic inflammation within the airway wall [19]. However, given the complex relationship between FeNO, eosinophils (particularly in sputum), and the influence of different treatments, these findings should be interpreted with caution and require validation in larger, well-characterized cohorts.

### 4.4. Prediction of Exacerbation Risk

BrWall_T2-MIS emerged as the sole independent predictor of high annual exacerbation rate (≥2 exacerbations per year, assessed over the 12 months following MRI) [15] within this pilot cohort in stepwise logistic regression analysis including pulmonary function tests, FeNO, and blood eosinophils as covariates, with an odds ratio of 1.11 (95% CI: 1.02–1.20) per unit increase in signal intensity [15]. This finding is clinically relevant and, albeit from a small pilot cohort, suggests potential utility in risk stratification and identification of patients who might benefit from treatment intensification.

It should be noted that the quantitative findings summarized in Table 1 derive predominantly from a single pilot cohort [15]; independent replication in larger, multicentre cohorts is needed before these estimates can be considered generalizable.

A summary of current evidence on BrWall_T2-MIS across all evaluated domains is provided in Table 2.

## 5. Comparison with CT-Derived Airway Metrics

A key question is whether BrWall_T2-MIS simply duplicates CT bronchial wall measurements. Evidence suggests these are partially independent [15,16].

The 2021 validation study showed MRI-UTE accurately measures bronchial dimensions and differentiates severe from non-severe asthma [16]. However, the 2025 T2-weighted study revealed important discordances: patients stratified by CT wall area percentage showed no significant BrWall_T2-MIS differences [15]. Notably, some patients with highest CT wall thickening did not show elevated T2 signal.

These observations suggest CT wall thickness reflects structural remodelling (smooth muscle hypertrophy, fibrosis), whereas T2-weighted signal captures active inflammation (edema, cellular infiltration, increased tissue water) [4]. This distinction matters clinically: remodelling represents fixed changes less responsive to anti-inflammatory therapy, whereas inflammation is potentially reversible with treatment.

## 6. Limitations

Current evidence is limited to single-centre studies, restricting generalizability [15]. The quantification workflow requires specialized software, and protocol standardization across scanners and centres remains lacking. Broader clinical adoption is further constrained by the cost of dedicated thoracic MRI protocols and the limited availability of advanced pulse sequences (UTE, T2w-PROPELLER), currently largely confined to specialized centres.

T2-weighted resolution (1.4 × 1.4 × 3 mm^3^ voxel size) remains substantially lower than that of CT (0.625 mm^3^) or UTE-MRI (1 mm^3^) [15]. Moreover, quantification has so far been restricted to third-generation bronchial pathways, limiting assessment of small airways, a recognized site of pathology in severe asthma [20]. No longitudinal or treatment-response data are currently available, and the cellular mechanisms underlying increased T2 signal remain incompletely understood.

Signal intensity is inherently dependent on vendor and field strength, and reproducibility across platforms has not yet been established, limiting multicentre pooling. Although advances in low-dose CT continue to narrow the radiation disadvantage of CT relative to MRI, they do not eliminate it.

## 7. Future Research Directions

Future priorities include standardized multicentre validation protocols and correlation with bronchoscopic and inflammatory biomarkers to better define the biological meaning of BrWall_T2-MIS [21].

Longitudinal studies assessing signal changes under biological therapies (anti-IL-5, anti-IL-4Rα, anti-TSLP, anti-IgE) are needed to evaluate its potential as a treatment endpoint [22]. Integration with functional MRI (hyperpolarized gas) and quantitative CT could enable comprehensive phenotyping [23,24]. Radiomics and AI-based approaches may further support multimodal prediction of treatment response and exacerbation risk [25,26,27,28,29], although large standardized datasets will be required [30,31,32,33,34,35,36,37]. Finally, extension to pediatric populations represents a radiation-free opportunity to investigate airway inflammation during lung development [38], although implementation will need to address higher and irregular respiratory rates, motion artefacts, and potential sedation requirements affecting registration accuracy.

## 8. Potential Clinical Implications in the Biological Therapy Era

- Phenotyping: Identify patients with high tissue inflammatory burden even when peripheral biomarkers are equivocal or corticosteroid-suppressed, enabling spatial assessment of bronchial inflammation known to be segmental in severe asthma.
- Assess treatment response: Provide objective evidence of inflammation reduction following biological therapy, potentially earlier than clinical improvements, and spatially localize treatment-refractory segments to inform targeted bronchial biopsies.
- Guide treatment de-escalation: Help identify candidates for safe step-down in patients achieving remission.

Biological therapies have transformed severe asthma management but have increased the need for biomarkers capable of improving patient selection and monitoring treatment response. Current algorithms rely on blood eosinophils, FeNO, and IgE—useful but incomplete markers that do not directly assess tissue inflammation [22,39,40].

BrWall_T2-MIS could potentially:

- Phenotype: Identify patients with high tissue inflammatory burden despite equivocal peripheral biomarkers or corticosteroid suppression, enabling spatial assessment of bronchial inflammation, which is known to be segmental in severe asthma.
- Assess treatment response: Provide objective evidence of inflammatory changes following biological therapy, potentially preceding clinical improvement, and identify treatment-refractory regions to guide further investigation.
- Guide treatment de-escalation: Help identify patients suitable for safe step-down after achieving remission.

However, these applications remain speculative pending validation. Current evidence supports BrWall_T2-MIS as a research tool for mechanistic studies and exploratory trials rather than for routine clinical decision-making.

## 9. Conclusions

Bronchial wall T2-weighted MRI signal intensity represents a promising investigational biomarker addressing an unmet need for radiation-free, repeatable assessment of airway pathology in severe asthma. Its apparent sensitivity to airway inflammation rather than fixed structural remodelling suggests potential value for monitoring disease activity and treatment response—applications in which radiation concerns have historically limited the role of CT. Current evidence also indicates that BrWall_T2-MIS and CT-derived wall thickness provide complementary rather than redundant information, supporting MRI as a radiation-free adjunct to, rather than a replacement for, CT.

At present, however, evidence remains limited to small, single-centre studies, and the biological and clinical significance of BrWall_T2-MIS requires further confirmation before clinical implementation. If validated in larger prospective studies, this imaging biomarker could expand the role of MRI beyond structural assessment, contributing to more comprehensive phenotyping and longitudinal evaluation of severe asthma in the era of precision medicine.

## Figures and Tables

**Table 1:** Comparison of computed tomography (CT) and ultra-short echo time magnetic resonance imaging (UTE-MRI) for structural airway assessment in severe asthma, highlighting technical characteristics, practical considerations, clinical applications, and current validation status.

Feature	CT	MRI (UTE + T2-Weighted)
Ionizing radiation	Yes	No
Spatial resolution	High (0.625 mm^3^ isotropic)	Lower (UTE: 1 mm^3^; T2-weighted: 1.4 × 1.4 × 3 mm^3^)
Typical acquisition time	Seconds (single breath-hold)	Minutes (UTE: 6–7 min; T2-weighted: 4–8 min; free-breathing)
Contrast agent required	No (unenhanced protocol)	No (unenhanced protocol)
Structural airway wall assessment	Validated, widely used	Validated, currently limited to proximal/segmental (3rd-generation) bronchi
Mucus plugging detection	Validated	Not yet established
Sensitivity to active inflammation	Limited (structural surrogate only)	Emerging evidence (T2-weighted signal intensity)
Suitability for longitudinal/repeated monitoring	Limited by cumulative radiation exposure	Well suited (no ionizing radiation)
Equipment/sequence availability	Widely available	Limited to centres with UTE and PROPELLER/BLADE capability; sequences used to date are vendor-specific prototypes rather than standard clinical packages
Cost	Lower; broadly reimbursed	Higher; specialized acquisition and post-processing
Current validation status	Established clinical and research tool	Investigational; evidence limited to pilot, single-centre studies

**Table 2:** Summary of key evidence on bronchial wall T2-weighted MRI signal in severe asthma. This table summarizes current findings on BrWall_T2-MIS, including its ability to differentiate disease severity, correlations with lung function and type-2 inflammation, prediction of exacerbation risk, and complementary value to CT-derived metrics, with the originating study specified for each finding. Limitations and future research directions are also highlighted to guide educational and investigational use.

Domain	Findings	Clinical/Research Implications
**Differentiation of severity**	BrWall_T2-MIS elevated in severe vs non-severe asthma (74 ± 12 vs. 49 ± 14 AU; *p* < 0.001) [15]	May identify high-inflammatory burden patients not captured by standard biomarkers
**Correlation with lung function**	Inverse correlation with FEV_1_ % predicted (r = −0.54) and FEV_1_/FVC (r = −0.44) [15]	Signal intensity reflects airflow limitation and disease burden
**Association with type-2 inflammation**	Moderate correlation with blood eosinophils (r = 0.52); no significant FeNO correlation [15]	Potential tissue-level marker of eosinophilic inflammation
**Exacerbation risk prediction**	Only independent predictor of ≥2 exacerbations/year in pilot cohort [15]	May support risk stratification and treatment intensification decisions
**Comparison with CT metrics**	Partially independent from CT wall area; T2 signal reflects active inflammation, CT reflects structural remodelling [15,16]	Complements CT, may guide inflammation-targeted therapies
**Limitations**	Small single-centre studies; limited resolution; complex workflow; no longitudinal or treatment-response data [15,16]	Further multicenter validation, standardization, and longitudinal studies needed
**Future directions**	Longitudinal monitoring, integration with biological therapy response, multiparametric MRI, AI/radiomics, pediatric studies	Educational and investigational tool; potential for precision medicine in severe asthma

## Data Availability

No new data were created or analyzed in this study. Data sharing is not applicable to this article.
